# Transrectal endoscopic resection of intraperitoneal gastrointestinal stromal tumor facilitated by incidental segmental absence of intestinal musculature

**DOI:** 10.1055/a-2665-7697

**Published:** 2025-08-20

**Authors:** Shuqian Hu, Yiyu Qiao, Xueting Zhang, Min Min, Yan Liu

**Affiliations:** 1 12538 The School of Medicine, Nankai University, Tianjin, China; 2 651943 Department of Gastroenterology, The First Medical Center of PLA General Hospital, Beijing, China

Segmental absence of intestinal musculature (SAIM) is a rare anomaly, usually causing neonatal intestinal perforation, and is exceedingly uncommon in adults 1 2 3 . We report a unique adult case leveraging SAIM to facilitate transrectal natural orifice transluminal endoscopic surgery (NOTES).

A 52-year-old woman undergoing routine colonoscopy incidentally had a movable mass (2.5 cm × 1.5 cm) near the ileocecal junction, characterized by a distinct white surface ( Fig. 1 **a** ). External hospital endoscopic ultrasound indicated origin possibly from the muscularis propria ( Fig. 1 **b** ). She had 20 years of well-controlled hypertension managed with telmisartan/hydrochlorothiazide, without abdominal symptoms.

**Fig. 1:**
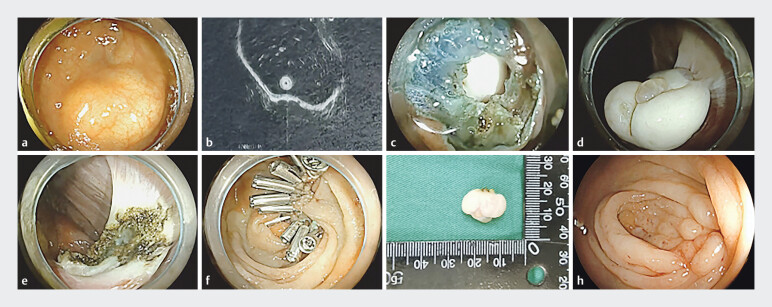
**a** Endoscopic view of mass; **b** muscularis propria origin suspected on EUS; **c** absence of muscular layers providing direct intraperitoneal access; **d** intraperitoneal white mass visualization; **e** electrocautery hemostasis; **f** defect closure with clips; **g** excised mass (2.0 cm × 1.5 cm); and **h** 3-month follow-up.

An initial attempt at endoscopic full-thickness resection revealed an unexpected SAIM, allowing direct intraperitoneal access immediately after submucosal dissection, without identifiable circular or longitudinal muscle layers ( Fig. 1 **c** ). This anatomical anomaly facilitated easy intraperitoneal access; a white mass was visualized intraperitoneally ( Fig. 1 **d** ), prompting a conversion to a transrectal NOTES approach. Excision was achieved and the intestinal defect was securely closed using five novel two-tooth clips (TTC) and six conventional clips ( Fig. 1 **e–g,** Fig. 2 , Video 1 ). Enhanced antibiotics were administered for 3 postoperative days, and oral intake resumed on day 3. Recovery was uneventful, with no abdominal pain, bleeding, or perforation.

**Fig. 2:**
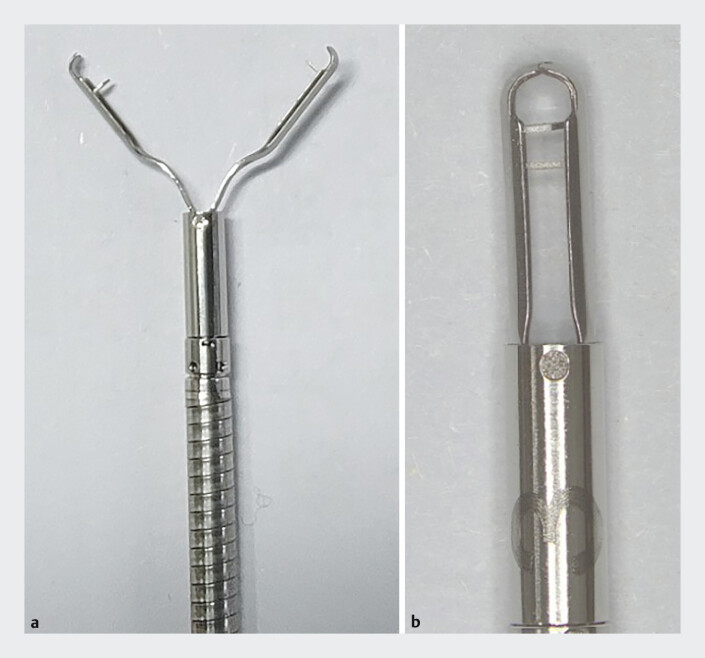
Novel two-tooth clip (TTC).

Transrectal natural orifice transluminal endoscopic resection of an intraperitoneal gastrointestinal stromal tumor facilitated by an incidental segmental absence of intestinal musculature.Video 1

Histopathology confirmed a gastrointestinal stromal tumor (GIST) consisting primarily of spindle cells with significant hyalinization, mild cytological atypia, and rare mitoses. At 3-month follow-up, the clips detached spontaneously ( Fig. 1 **h** ). Retrospective analysis confirmed SAIM, possibly congenital or related to hypertension 4 5 .

This case uniquely demonstrates the successful transrectal NOTES resection of an intraperitoneal GIST enabled by SAIM, highlighting the safe application of novel closure clips. Gastroenterologists and endoscopists should remain aware of SAIM as a potential incidental finding that could impact surgical decision-making, particularly concerning lesion accessibility and procedural adaptation.

Endoscopy_UCTN_Code_CPL_1AJ_2AD_3AD

## Notes

Endoscopy E-Videos https://eref.thieme.de/e-videos
*E-Videos* is an open access online section of the journal *Endoscopy* , reporting on interesting cases and new techniques in gastroenterological endoscopy. All papers include a high-quality video and are published with a Creative Commons CC-BY license. Endoscopy E-Videos qualify for HINARI discounts and waivers and eligibility is automatically checked during the submission process. We grant 100% waivers to articles whose corresponding authors are based in Group A countries and 50% waivers to those who are based in Group B countries as classified by Research4Life (see: https://www.research4life.org/access/eligibility/ ). This section has its own submission website at https://mc.manuscriptcentral.com/e-videos .
